# Illusory Increases in Font Size Improve Letter Recognition

**DOI:** 10.1177/0956797617705391

**Published:** 2017-07-05

**Authors:** Martin Lages, Stephanie C. Boyle, Rob Jenkins

**Affiliations:** 1 School of Psychology, University of Glasgow; 2 Institute of Neuroscience and Psychology, University of Glasgow; 3 Department of Psychology, University of York

**Keywords:** motion aftereffect, visual acuity, object recognition, size illusion, open data

## Abstract

Visual performance of human observers depends not only on the optics of the eye and early sensory encoding but also on subsequent cortical processing and representations. In two experiments, we demonstrated that motion adaptation can enhance as well as impair visual acuity. Observers who experienced an expanding motion aftereffect exhibited improved letter recognition, whereas observers who experienced a contracting motion aftereffect showed impaired letter recognition. We conclude that illusory enlargement and shrinkage of a visual stimulus can modulate visual acuity.

Visual acuity is commonly assessed using a logarithm-of-the-minimum-angle-of-resolution (LogMAR) chart (Bailey & Lovie, 1976; Ferris, Kassoff, Bresnick, & Bailey, 1982). The chart contains letters in unpredictable sequences; these sequences are arranged in horizontal rows, and the rows are stacked vertically. Letters in successive rows are printed at smaller sizes, so that they are increasingly difficult to read. The observer’s task is to read as far down the chart as possible while maintaining a fixed viewing distance. Visual acuity is calculated from the size at which the observer can no longer identify the letters reliably (Arditi & Cagenello, 1993; Snellen, 1862).

This method is based on a simple principle: Visual stimuli are easier to resolve when they are large than when they are small, because a larger image carries more detail. This principle is uncontroversial for physical changes in image size, for example, when letter size increases or viewing distance decreases. However, neuroimaging studies have revealed parallels between apparent size and physical size at the level of cortical representation. In particular, the perceived size of a stimulus can modulate the spatial extent of its representation in V1, even when physical stimulus size is held constant (Fang, Boyaci, Kersten, & Murray, 2008; Murray, Boyaci, & Kersten, 2006; Ni, Murray, & Horwitz, 2014; Sperandio, Chouinard, & Goodale, 2012).

One method of altering perceived stimulus size while maintaining physical stimulus size is through prolonged adaptation to spiral motion (Holland, 1965; Thompson, 1880). If an observer adapts to contracting or expanding motion of a rotating spiral, a subsequent static image appears to expand or contract in the opposite direction around fixation (Lages, Adams, & Graf, 2009). This well-established phenomenon is known as the *spiral motion aftereffect*—a specific version of the motion aftereffect (Addams, 1834; Anstis, Verstraten, & Mather, 1998). The spiral motion aftereffect allows one to study consequences of perceived image size without having to manipulate physical image size or viewing distance (Kersten & Murray, 2010; Schindel & Arnold, 2010; Sloan, 1951). For example, it allows one to ask whether illusory enlargement of letters can enhance their identification.

In two experiments, we assessed the impact of perceived stimulus size on visual acuity after motion adaptation. In Experiments 1a and 1b, we employed letters with different but fixed font sizes in a between-subjects design, and in Experiment 2, we used adaptive font sizes in a within-subjects design. Our results demonstrate that the motion aftereffect can modulate visual acuity. More specifically, illusory expanding motion can improve visual acuity, while illusory contracting motion can decrease visual acuity.

## General Method

Both of the experiments adhered to the guidelines of the Declaration of Helsinki. An initial power analysis suggested that a sample size between 30 and 46 observers would achieve the desired effect size (Cohen’s *f* = 0.20–0.25, α = .05, β = 0.90). Participants were students from the University of Glasgow between the ages of 16 and 28 years. Only participants who had normal or corrected-to-normal visual acuity of 10/10 (0.0 logMAR) on a logarithmic visual-acuity chart (Catalog No. 2103, Precision Vision, Woodstock, IL) took part in the experiments.

The stimulus and task were programmed in MATLAB (The MathWorks, Natick, MA) using the Psychophysics Toolbox extensions (Brainard, 1997; Pelli, 1997). In both experiments, we used Helvetica bold capital as the text font for the Sloan letters C, D, H, K, N, O, R, S, V, and Z (Sloan, 1951). These letters were randomly selected and anti-aliased. Letters were displayed on screen at a viewing distance of 3 m (10 feet) in a dark room with the lights switched off.

**Fig. 1.**
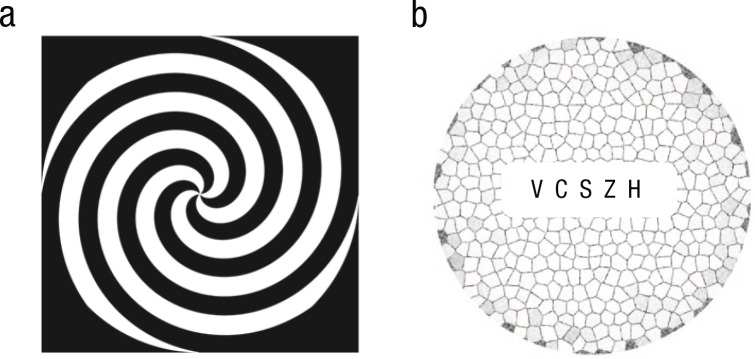
Illustration of (a) a linear spiral with high-contrast segments, a moving version of which was presented in the adaptation phase, and (b) an example test stimulus consisting of five Sloan letters surrounded by a Voronoi pattern. In the adaptation phase, spirals rotated either clockwise (inducing adaptation to contracting motion) or counterclockwise (inducing adaptation to expanding motion). Sloan letters ranged in size from −0.3 to 0.0 on the logarithm-of-the-minimum-angle-of-resolution (logMAR) chart (Snellen fraction: 10/5–10/10) and were surrounded by a Voronoi pattern.

Each observer completed a preadaptation and an adaptation block. In the preadaptation block, a test image with five horizontally aligned letters was presented for 6 s, and the observer was asked to read out each letter from left to right. An experimenter recorded each response (whether correct or incorrect). The five letters were separated horizontally by double spaces and surrounded by a circular Voronoi pattern on a white background (Fig. 1b). The Voronoi pattern consisted of randomly oriented lines that elicited a strong contracting or expanding motion aftereffect in the test stimulus after motion adaptation.

In the adaptation block, the same test stimuli were used, but each test stimulus was preceded by a 30-s motion-adaptation phase. During motion adaptation, the observer fixated on the center of a black and white linear spiral (see Fig. 1a) that rotated clockwise or counterclockwise at a speed of 90° per second. Depending on the direction of rotation, observers adapted to contracting or expanding motion so that the ensuing motion aftereffect created illusory expansion or contraction in the test images.

## Experiments 1a and 1b

### Method

In Experiments 1a and 1b, we recruited 45 and 35 participants, respectively. One participant in Experiment 1a and 5 participants in Experiment 1b had to be excluded because they did not meet our requirement of 10/10 visual acuity or were tested under different lighting conditions from the other participants. This left data from 44 observers (16 male, 28 female) in Experiment 1a and 30 observers (11 male, 19 female) in Experiment 1b for analysis. Each observer completed a preadaptation and an adaptation block of 16 trials each. A block featured four sets of trials with letters in different font sizes. The largest font size was used in the first set of trials, followed by increasingly smaller font sizes in the next three sets. Five letters of the same font size were presented in each of the four trials of a set. Thus, an observer had to identify a total of 80 letters (5 letters × 4 sets × 4 trials) per block, with 20 letters at each font size. The four font sizes subtended 4.9, 4.0, 3.1, and 2.2 minutes of arc (arcmin), respectively. Recognizing 4 out of 5 letters at a given size approximated Snellen fractions of 10/10 (0.0 logMAR), 10/8 (−0.1 logMAR), 10/6.3 (−0.2 logMAR), and 10/5 (−0.3 logMAR), respectively. Adaptation and test stimuli subtended 2.2 degrees of visual angle on a 21-in. CRT monitor (Vision Master Pro 514, Iiyama, Tokyo, Japan) with a resolution of 0.32 mm per pixel at a refresh rate of 120 Hz.

In Experiment 1a, we sought to determine whether adaptation to contracting motion followed by an expanding motion aftereffect would improve visual acuity. We measured the number of letters that the 44 observers correctly recognized at the four different font sizes, first in the preadaptation block of trials and again after adaptation to contracting motion in the second block of trials. As expected, all observers passed the acuity test for the largest letter size (0.0 logMAR) in the preadaptation block, achieving a Snellen fraction of 10/10 or better.

Conversely, in Experiment 1b, we sought to determine whether adapting to expanding motion followed by a contracting motion aftereffect would impair visual acuity. As in Experiment 1a, we measured the number of correctly recognized letters at four different font sizes, first in a preadaptation block and again after adaptation to expanding motion in a second block. All 30 observers achieved a visual acuity of at least 10/10 in the preadaptation block.

### Results

First, we conducted separate analyses of variance (ANOVAs) on the number of correct letter recognitions in Experiments 1a and 1b, respectively. The within-subjects factors were adaptation (preadaptation, adaptation to contracting motion in Experiment 1a; preadaptation, adaptation to expanding motion in Experiment 1b), font size (−0.3, −0.2, −0.1, 0.0 logMAR), and letter position (1–5 from left to right). In order to compare performance change at different levels of baseline acuity, we introduced a between-subjects factor by dividing observers into two groups on the basis of their preadaptation performance: one with normal visual acuity and one with high visual acuity.

**Fig. 2.**
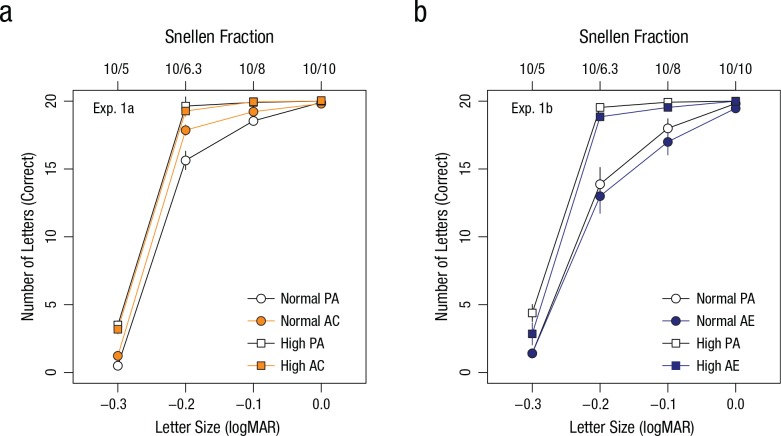
Results of (a) Experiment 1a and (b) Experiment 1b: mean number of correctly identified letters as a function of font size, separately for each combination of block and performance group (normal vs. high, based on a median split). Observers first completed a preadaptation (PA) block, followed by a block in which they either adapted to contracting motion (AC; Experiment 1a) or adapted to expanding motion (AE; Experiment 1b). Error bars denote ±1 *SEM*, and in some cases are smaller than the data points. logMAR = logarithm of the minimum angle of resolution.

Figure 2a illustrates observers’ performance in Experiment 1a plotted against font size. We used a median split to categorize observers as having either normal or high visual acuity. We observed a statistically significant main effect between the two groups, *F*(1, 42) = 30.8, *p* < .001, η_*p*_^2^ = .42, which confirmed contrasting levels of baseline performance for observers below and above the sample median. There was also a main effect of font size, *F*(3, 126) = 1,705.7, *p* < .001, η_*p*_^2^ = .98, which reflects the steep increase in performance with font size.

The ANOVA on letter recognition in Experiment 1b also revealed a statistically significant effect between groups, *F*(1, 28) = 17.6, *p* < .001, η_*p*_^2^ = .39, and font size, *F*(3, 126) = 481.7, *p* < .001, η_*p*_^2^ = .95 (Fig. 2b); this confirms that there were different levels of performance between observers with normal and high baseline acuity and an increasing difficulty to recognize smaller letters.

Critically for our hypothesis, results showed that adaptation to contracting motion in Experiment 1a resulted in statistically significant improvements, *F*(1, 42) = 7.80, *p* = .0078, η_*p*_^2^ = .16, when comparing performance in the preadaptation and adaptation blocks. Significant interactions between adaptation and group, *F*(1, 42) = 12.73, *p* = .001, and among adaptation, font size, and group, *F*(3, 126) = 6.98, *p* = .001, revealed that the adaptation effect in Experiment 1a was due to observers with normal visual acuity who identified on average 11% (2.2 out of 20) more letters at −0.2 logMAR and also showed small improvements at −0.3 and −0.1 logMAR. Observers with high visual acuity showed no significant improvement for any of the font sizes (Fig. 2a).

Importantly, adaptation to expanding motion in Experiment 1b had the opposite effect: We found a small but statistically significant reduction in performance, *F*(1, 28) = 8.01, *p* = .0085, η_*p*_^2^ = .22, when comparing the preadaptation and adaptation blocks. This time, however, there were no significant interactions qualifying the adaptation effect. On average, observers with high visual acuity recognized 7.5% (1.5 out of 20) fewer letters at −0.3 logMAR after motion adaptation, and observers with normal visual acuity recognized an average of 5% (1.0 out of 20) fewer letters at −0.1 logMAR (Fig. 2b).

We summarized performance across different font sizes by estimating individual thresholds for letter recognition. Separately for each experiment, we fitted cumulative Gaussian functions (Wichmann & Hill, 2001) to the individual data from each block, derived the 50% thresholds for the preadaptation and adaptation block, and computed the difference between those thresholds. Comparing the threshold differences in each group revealed a statistically significant change in performance (*M* = −0.41 arcmin, 95% confidence interval = [–0.61, –0.21]), two-samples *t*(63) = −4.1, *p* = .0001.

**Fig. 3.**
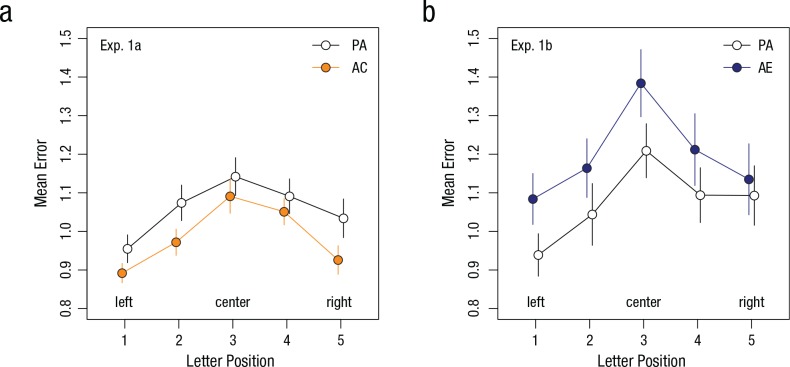
Results of (a) Experiment 1a and (b) Experiment 1b: mean error as a function of letter position and block. Mean error was defined as the number of unrecognized letters averaged across font sizes and observers. Observers completed two blocks: preadaptation (PA) and either adaptation to contracting motion (AC; Experiment 1a) or adaptation to expanding motion (AE; Experiment 1b). Error bars denote ±1 *SEM*.

Figure 3 illustrates the significant main effects of letter position in Experiment 1a, *F*(4, 168) = 9.42, *p* < .0001, η_*p*_^2^ = .18, and in Experiment 1b, *F*(4, 112) = 9.86, *p* < .001, η_*p*_^2^ = .26. The letter-position effect indicates that letters at the center of the display attracted significantly more errors compared with letters on the left and right. The significant effects of letter position suggest a form of foveal crowding in which flanking letters impair recognition of letters at the center (Lev, Yehezkel, & Polat, 2014; Levi, Klein, & Aitsebaomo, 1985). However, we found no significant interaction between adaptation and letter position in Experiments 1a and 1b (*F*s < 1). This indicated that crowding and illusory motion affected letter recognition independently of each other.

**Fig. 4.**
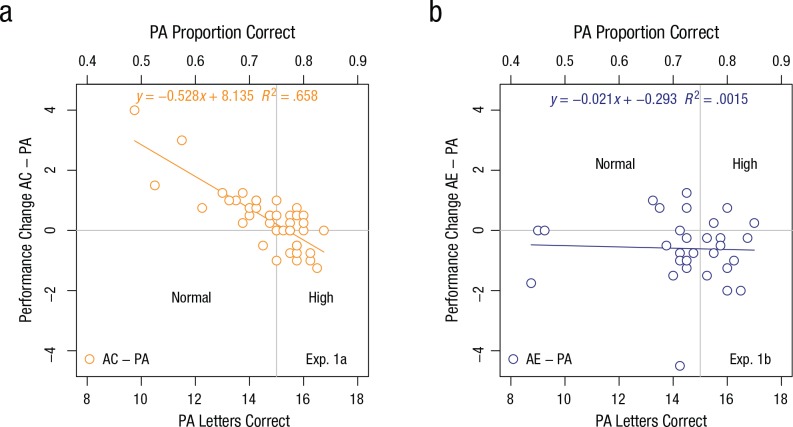
Results of (a) Experiment 1a and (b) Experiment 1b: scatterplots (with best-fitting regression lines) showing the relationship between preadaptation (PA) performance and change in performance between the PA block and the adaptation block. Observers adapted to either contracting motion (AC; Experiment 1a) or expanding motion (AE; Experiment 1b). In both plots, PA performance is expressed as both the mean number and the proportion of correctly identified letters. The vertical line splits observers into groups with normal and high PA visual acuity. The horizontal line separates observers with improved performance from observers with impaired performance.

In Figures 4a and 4b, visual acuity in preadaptation is plotted against performance change for each observer in Experiment 1a and Experiment 1b, respectively. Performance change is expressed as the difference between correctly identified letters in the adaptation and preadaptation conditions. Figure 4a shows a negative association between initial visual acuity and performance change resulting from the expanding motion aftereffect (Pearson’s *r* = −0.81, *p* < .001; *R*^2^ = .658). Together with Figure 2a, this suggests that observers with high visual acuity did not benefit from the expanding motion aftereffect, whereas observers with normal visual acuity showed improved acuity at font sizes −0.1 and −0.2 logMAR.

In contrast to Experiment 1a, there was reduced visual acuity as a consequence of the contracting motion aftereffect in Experiment 1b and no significant association between initial acuity and performance change (Fig. 4b; Pearson’s *r* = −.039, *p* = .838; *R*^2^ = .0015). In an additional ANOVA with mixed effects, we combined the data from Experiments 1a and 1b (*N* = 74) and entered change in performance (adaptation block – preadaptation block) as the dependent variable. Font size and letter position were within-subjects factors, and experiment (1a, 1b) was a between-subjects factor. The results confirmed that adaptation to contracting and expanding motion produced the only statistically significant effect on change in performance, *F*(1, 72) = 15.1, *p* < .001, η_*p*_^2^ = .17.

## Experiment 2

### Method

In a follow-up experiment, we varied motion adaptation within subjects in separate sessions over consecutive days. A total of 41 naive students from the University of Glasgow were recruited, but 3 students did not meet the requirement of 10/10 visual acuity, and a further 6 did not complete the second session. As a result, data of 32 observers (age range = 19–23 years; 12 male, 20 female) were available for analysis. Instead of fixed font sizes, we employed an adaptive staircase method to determine individual thresholds of letter size (Watson & Pelli, 1983). As a consequence, letter size varied from trial to trial depending on previous responses and letter size. Stimuli were displayed on a high-resolution 27-in. LED monitor (Cinema Display, Apple, Cupertino, CA) with a resolution of 0.23 mm per pixel at a refresh rate of 60 Hz and mean luminance of 89 cd/m^2^.

Each observer attended two sessions on consecutive days at the same time of day. Each session consisted of a preadaptation and an adaptation block of 40 trials each. Observers adapted to one form of motion in the first session and the other form of motion in the following session; the sequence of the adaptation conditions (adaptation to contracting motion first, adaptation to expanding motion first) was counterbalanced across participants to control for possible carryover effects.

### Results

The differences between threshold estimates from the preadaptation and adaptation blocks on each day were entered into a two-way ANOVA with session (Session 1, Session 2) and sequence (adaptation to contracting motion first, adaptation to expanding motion first) as factors. A negative threshold difference indicated improved performance, and a positive threshold difference indicated impaired individual performance. The analysis produced a statistically significant interaction between session and sequence, *F*(1, 30) = 5.22, *p* = .03, η_*p*_^2^ = .15.

**Fig. 5.**
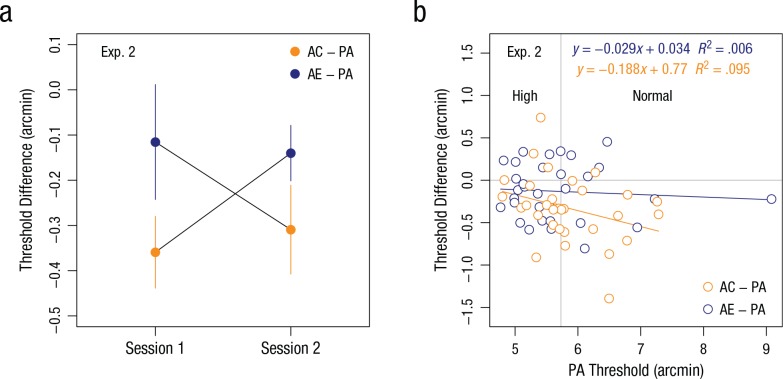
Results of Experiment 2. The graph (a) shows the difference in letter-recognition thresholds between the preadaptation (PA) and adaptation blocks at each session, separately for observers who adapted to contracting motion (AC) first and observers who adapted to expanding motion (AE) first. Error bars denote ±1 *SEM*. The scatterplot (b; with best-fitting regression line) shows the relationship between the PA letter-recognition threshold and the threshold difference between the PA and adaptation blocks, separately for observers in the AC and AE groups. The vertical line splits observers into groups with normal and high PA visual acuity. The horizontal line separates observers with improved performance from observers with impaired performance.

The interaction shown in Figure 5a illustrates the expected opposite adaptation effects for the two sequences: The average threshold difference for adaptation to contracting motion in Session 1 is lower compared with adaptation to expanding motion in Session 2 for observers who adapted to contracting motion prior to adapting to expanding motion. Similarly, the average threshold difference for adaptation to expanding motion in Session 1 was higher compared with adaptation to contracting motion in Session 2 for observers who adapted to expanding motion in the first session. In Figure 5b, thresholds of preadaptation are plotted against threshold differences between adaptation and preadaptation blocks for each observer. Despite a sizeable overlap between the two adaptation conditions, there was a statistically significant change in performance between adaptation to expanding motion and contracting motion (*M* = −0.20 arcmin, 95% confidence interval = [–0.37, –0.03]); one-sample *t*(31) = −2.28, *p* = .029.

## Discussion

The present findings convincingly demonstrate that visual acuity can be modulated by previous adaptation to contracting or expanding motion. In Experiment 1a, the expanding motion aftereffect improved letter recognition in observers with normal visual acuity, whereas observers with high visual acuity performed at ceiling and could not improve further. In Experiment 2, individual observers adapted to contracting as well as expanding motion in separate sessions. Using an adaptive staircase method in a within-subjects design, we confirmed the differential effect of motion adaptation on letter recognition. In particular, illusory expansion in the test stimulus helped identification of smaller letters that were difficult to discern otherwise.

What is the most plausible explanation for the effect of motion adaptation on visual acuity? We can rule out pupil size as a confounding factor (Kloosterman et al., 2015; Laeng & Endestad, 2012), since the test stimuli were considerably brighter than the adaptation stimulus: Luminance of the test stimulus measured on screen changed by 41 cd/m^2^ in Experiment 1 and by 92 cd/m^2^ in Experiment 2. In a control study (*N* = 6), we confirmed that all observers’ pupils contracted immediately after onset of the test stimulus in all conditions, but there was no opposite effect on pupil size during the expanding and contracting motion aftereffect. Other possible explanations, such as adaptation to blur (Mon-Williams, Tresilian, Strang, Kochhar, & Wann, 1998; Pseudovs & Brennan, 1993; Webster, Georgeson, & Webster, 2002) and motion blur (Barlow & Olshausen, 2004) did not predict opposite effects on performance as found in Experiments 1 and 2.

It is possible that the motion aftereffect shifted and scaled the representation of the stimulus in cortical area V1 (Murray et al., 2006; Ni et al., 2014; Sperandio et al., 2012; Whitney et al., 2003). More specifically, the motion aftereffect may have modulated surround suppression in the retinotopically organized receptive fields of the medial temporal brain region (Anton-Erxleben, Stephan, & Treue, 2009), which possibly altered spatial resolution at earlier processing stages through recurrent feedback (Anton-Erxleben & Carrasco, 2013; Carrasco, Williams, & Yeshurun, 2002; Fang et al., 2008). Although this explanation is rather speculative (e.g., Morgan, 2012), a more extensive neural representation in retinotopically organized cortical areas would make it easier to resolve discriminating features during object recognition (Barlow, 1961; Vinje & Gallant, 2000).

Another possible explanation is that the expanding motion aftereffect did not alter the neural representation but facilitated the “readout” or recognition of letters. The contracting motion aftereffect may have increased foveal crowding, whereas the expanding motion aftereffect may have released crowding between letters and the surrounding Voronoi pattern (Lev et al., 2014; Levi et al., 1985). This would help and impede recognition of letters, respectively (Herzog, Sayim, Chicherov, & Manassi, 2015). Although the position effect in Experiments 1a and 1b is reminiscent of the effect of crowding between letters in the periphery (Bouma, 1970; Dakin, Greenwood, Carlson, & Bex, 2011; Falkenberg, Rubin, & Bex, 2007; Pelli & Tillman, 2008; Whitney & Levi, 2011), we found no significant interaction between adaptation and letter position. This suggests that any crowding effect was independent of the motion aftereffect.

We conclude that an illusory change in perceived stimulus size after motion adaptation is responsible for this small but surprising effect. Visual acuity is routinely associated with the optics of the human eye and refractive errors. The striking implication of our findings is that, under the conditions described here, adaptation to contracting motion can improve visual acuity.

## Supplementary Material

Supplementary material

## References

[bibr1-0956797617705391] Addams R. (1834). An account of a peculiar optical phenomenon seen after having looked at a moving body, etc. London and Edinburgh Philosophical Magazine and Journal of Science, 5, 373–374. doi:10.1080/14786443408648481

[bibr2-0956797617705391] Anstis S. Verstraten F. A. J. Mather G. (1998). The motion aftereffect. Trends in Cognitive Sciences, 2, 111–117. doi:10.1016/S1364-6613(98)01142-5 21227087

[bibr3-0956797617705391] Anton-Erxleben K. Carrasco M. (2013). Attentional enhancement of spatial resolution: Linking behavior and neurophysiological evidence. Nature Reviews Neuroscience, 14, 188–200. doi:10.1038/nrn3443 23422910 PMC3977878

[bibr4-0956797617705391] Anton-Erxleben K. Stephan V. M. Treue S. (2009). Attention reshapes center-surround receptive field structure in macaque cortical area MT. Cerebral Cortex, 19, 2466–2478. doi:10.1093/cercor/bhp002 19211660 PMC2742598

[bibr5-0956797617705391] Arditi A. Cagenello R. (1993). On the statistical reliability of letter-chart visual acuity measurements. Investigative Ophthalmology & Visual Science, 34, 120–129. 8425819

[bibr6-0956797617705391] Bailey I. L. Lovie J. E. (1976). New design principles for visual acuity letter charts. American Journal of Optometry and Physiological Optics, 53, 740–745. doi:10.1097/00006324-197611000-00006 998716

[bibr7-0956797617705391] Barlow H. B. (1961). Possible principles underlying the transformations of sensory messages. In Rosenblith W. A. (Ed.), Sensory communication (pp. 217–234). Cambridge, MA: MIT Press.

[bibr8-0956797617705391] Barlow H. B. Olshausen B. A. (2004). Convergent evidence for the visual analysis of optic flow through anisotropic attenuation of high spatial frequencies. Journal of Vision, 4(6), 415–426. doi:10.1167/4.6.1 15330709

[bibr9-0956797617705391] Bouma H. (1970). Interaction effects in parafoveal letter recognition. Nature, 226, 177–178. doi:10.1038/226177a0 5437004

[bibr10-0956797617705391] Brainard D. H. (1997). The Psychophysics Toolbox. Spatial Vision, 10, 433–436. doi:10.1163/156856897X00357 9176952

[bibr11-0956797617705391] Carrasco M. Williams P. E. Yeshurun Y. (2002). Covert attention increases spatial resolution with or without masks: Support for signal enhancement. Journal of Vision, 2(6), 467–479. doi:10.1167/2.6.4 12678645

[bibr12-0956797617705391] Dakin S. C. Greenwood J. A. Carlson T. A. Bex P. J. (2011). Crowding is tuned for perceived (not physical) location. Journal of Vision, 11(9), Article 2. doi:10.1167/11.9.2 PMC3627388 21824980

[bibr13-0956797617705391] Falkenberg H. K. Rubin G. S. Bex P. J. (2007). Acuity, crowding, reading and fixation stability. Vision Research, 47, 126–135. doi:10.1016/j.visres.2006.09.014 17078991

[bibr14-0956797617705391] Fang F. Boyaci H. Kersten D. Murray S. O. (2008). Attention-dependent representation of a size illusion in human V1. Current Biology, 18, 1707–1712. doi:10.1016/j.cub.2008.09.025 18993076 PMC2638992

[bibr15-0956797617705391] Ferris F. L. III Kassoff A. Bresnick G. H. Bailey I. (1982). New visual acuity charts for clinical research. American Journal of Ophthalmology, 94, 91–96. doi:10.1016/0002-9394(82)90197-0 7091289

[bibr16-0956797617705391] Herzog M. H. Sayim B. Chicherov V. Manassi M. (2015). Crowding, grouping, and object recognition: A matter of appearance. Journal of Vision, 15(6), Article 5. doi:10.1167/15.6.5 PMC4429926 26024452

[bibr17-0956797617705391] Holland H. C. (1965). The spiral after-effect. London, England: Pergamon Press Ltd.

[bibr18-0956797617705391] Kersten D. Murray S. O. (2010). Vision: When does looking bigger mean seeing better? Current Biology, 20, R398–R399. doi:10.1016/j.cub.2010.03.021 20462478

[bibr19-0956797617705391] Kloosterman N. A. Meindertsma T. van Loon A. M. Lamme V. A. F. Bonneh Y. S. Donner T. H. (2015). Pupil size tracks perceptual content and surprise. European Journal of Neuroscience, 41, 1068–1078. doi:10.1111/ejn.12859 25754528

[bibr20-0956797617705391] Laeng B. Endestad T. (2012). Bright illusions reduce the eye’s pupil. Proceedings of the National Academy of Sciences, USA, 109, 2162–2167. doi:10.1073/pnas.1118298109 PMC3277565 22308422

[bibr21-0956797617705391] Lages M. Adams W. J. Graf E. W. (2009). Motion-aftereffect-induced blindness. Journal of Vision, 9(11), Article 11. doi:10.1167/9.11.11 20053074

[bibr22-0956797617705391] Lev M. Yehezkel O. Polat U. (2014). Uncovering foveal crowding? Scientific Reports, 4, Article 4067. doi:10.1038/srep04067 PMC3921636 24518803

[bibr23-0956797617705391] Levi D. M. Klein S. A. Aitsebaomo A. P. (1985). Vernier acuity, crowding and cortical magnification. Vision Research, 25, 963–977. doi:10.1016/0042-6989(85)90207-X 4049746

[bibr24-0956797617705391] Mon-Williams M. Tresilian J. R. Strang N. C. Kochhar P. Wann J. P. (1998). Improving vision: Neural compensation for optical defocus. Proceedings of the Royal Society B: Biological Sciences, 265, 71–77. doi:10.1098/rspb.1998.0266 PMC1688761 9470217

[bibr25-0956797617705391] Morgan M. J. (2012). Motion adaptation does not depend on attention to the adaptor. Vision Research, 55, 47–51. doi:10.1016/j.visres.2011.12.009 22245710 PMC4135072

[bibr26-0956797617705391] Murray S. O. Boyaci H. Kersten D. (2006). The representation of perceived angular size in human primary visual cortex. Nature Neuroscience, 9, 429–434. doi:10.1038/nn1641 16462737

[bibr27-0956797617705391] Ni A. M. Murray S. O. Horwitz G. D. (2014). Object-centered shifts of receptive field positions in monkey primary visual cortex. Current Biology, 24, 1653–1658. doi:10.1016/j.cub.2014.06.003 25017208 PMC4123419

[bibr28-0956797617705391] Pelli D. G. (1997). The VideoToolbox software for visual psychophysics: Transferring numbers into movies. Spatial Vision, 10, 437–442. doi:10.1163/156856897X00366 9176953

[bibr29-0956797617705391] Pelli D. G. Tillman K. A. (2008). The uncrowded window of object recognition. Nature Neuroscience, 11, 1129–1135. doi:10.1038/nn1208-1463b 18828191 PMC2772078

[bibr30-0956797617705391] Pseudovs K. Brennan N. A. (1993). Decreased uncorrected vision after a period of distance fixation with spectacle wear. Optometry and Vision Science, 70, 528–531. doi:10.1097/00006324-199307000-00002 8355963

[bibr31-0956797617705391] Schindel R. Arnold D. H. (2010). Visual sensitivity can scale with illusory size changes. Current Biology, 20, 841–844. doi:10.1016/j.cub.2010.02.068 20434339

[bibr32-0956797617705391] Sloan L. L. (1951). Measurement of visual acuity: A critical review. Archives of Ophthalmology, 45, 704–725. doi:10.1001/archopht.1951.01700010719013 14902186

[bibr33-0956797617705391] Snellen H. (1862). Probebuchstaben zur Bestimmung der Sehschärfe [Sample letters for the determination of visual acuity]. Utrecht, The Netherlands: Van de Weijer.

[bibr34-0956797617705391] Sperandio I. Chouinard P. A. Goodale M. A. (2012). Retinotopic activity in V1 reflects the perceived and not the retinal size of an afterimage. Nature Neuroscience, 15, 540–542. doi:10.1038/nn.3069 22406550

[bibr35-0956797617705391] Thompson P. (1880). Optical illusions of motion. Brain, 3, 289–298.

[bibr36-0956797617705391] Vinje W. E. Gallant J. L. (2000). Sparse coding and decorrelation in primary visual cortex during natural vision. Science, 287, 1273–1276. doi:10.1126/science.287.5456.1273 10678835

[bibr37-0956797617705391] Watson A. B. Pelli D. G. (1983). QUEST: A Bayesian adaptive psychometric method. Perception & Psychophysics, 33, 113–120. doi:10.3758/BF03202828 6844102

[bibr38-0956797617705391] Webster M. A. Georgeson M. A. Webster S. M. (2002). Neural adjustments of image blur. Nature Neuroscience, 5, 839–840. doi:10.1167/1.3.441 12195427

[bibr39-0956797617705391] Whitney D. Goltz H. C. Thomas C. G. Gati J. S. Menon R. S. Goodale M. A. (2003). Flexible retinotopy: Motion-dependent position coding in the visual cortex. Science, 302, 878–881. doi:10.1126/science.1087839 14500849 PMC3849414

[bibr40-0956797617705391] Whitney D. Levi D. M. (2011). Visual crowding: A fundamental limit on conscious perception and object recognition. Trends in Cognitive Sciences, 15, 160–168. doi:10.1016/j.tics.2011.02.005 21420894 PMC3070834

[bibr41-0956797617705391] Wichmann F. A. Hill N. J. (2001). The psychometric function: I. Fitting, sampling, and goodness of fit. Perception & Psychophysics, 63, 1293–1313. doi:10.3758/BF03194544 11800458

